# Alkyl Hydroperoxide Reductase as a Determinant of Parasite Antiperoxide Response in *Toxoplasma gondii*

**DOI:** 10.1155/2021/1675652

**Published:** 2021-09-21

**Authors:** Jinwen Wang, Qianqian Tan, Junpeng Chen, Xiaomei Liu, Zeyan Di, Qianqian Xiao, Jinxuan Li, Xiaomin Zhao, Xiao Zhang

**Affiliations:** ^1^Department of Preventive Veterinary Medicine, College of Veterinary Medicine, Shandong Agricultural University, Tai'an, China; ^2^Shandong Provincial Key Laboratory of Animal Biotechnology and Disease Control and Prevention, Shandong Agricultural University, Tai'an, China; ^3^Shandong Provincial Engineering Technology Research Center of Animal Disease Control and Prevention, Shandong Agricultural University, Tai'an, China

## Abstract

*Toxoplasma gondii* is a protozoan parasite that is widely parasitic in the nucleated cells of warm-blooded animals. Bioinformatic analysis of alkyl hydroperoxide reductase 1 (AHP1) of *T. gondii* is a member of the Prxs family and exhibits peroxidase activity. Cys^166^ was certified to be a key enzyme active site of TgAHP1, indicating that the enzyme follows a cysteine-dependent redox process. TgAHP1 was present in a punctate staining pattern anterior to the *T. gondii* nucleus. Oxidative stress experiments showed that the ∆Ahp1 strain was more sensitive to tert-butyl hydroperoxide (tBOOH) than hydrogen peroxide (H_2_O_2_), indicating that tBOOH may be a sensitive substrate for TgAHP1. Under tBOOH culture conditions, the ∆Ahp1 strain was significantly less invasive, proliferative, and pathogenic in mice. This was mainly due to the induction of tBOOH, which increased the level of reactive oxygen species in the parasites and eventually led to apoptosis. This study shows that TgAHP1 is a peroxisomes protein with cysteine-dependent peroxidase activity and sensitive to tBOOH.

## 1. Introduction

*T. gondii* is an obligate intracellular parasitic protozoan that infects a wide variety of warm-blooded animals [1], which are widely distributed across the world, both in terms of their geographical location and the diversity of their host range. Infection by this parasite causes congenital diseases and abortions in farm animals, which can lead to significant reproductive losses and consequent economic losses [2].

In most eukaryotes, oxygen is usually required for the participation of normal cellular physiological functions. This part of oxygen ions is partially reduced to superoxide anions and then reduced to other reactive oxygen species (ROS) [3], such as hydrogen peroxide and hydroxyl radicals [4]. In the early stages of parasite invasion, macrophages and natural killer cells mainly transmit reactive oxygen species (ROS) to eliminate *T. gondii* [5]. For the elimination of ROS, the pathogen had a specific ROS detoxification mechanism. It has been detected that protozoa contain some major antioxidant enzymes, which are the main components of the enzymatic detoxification pathway and act downstream of superoxide dismutase (SOD) to prevent the formation of hydroxyl radicals by detoxifying H_2_O_2_. The low concentration of ROS participates in the cellular signal transduction process. However, superabundant ROS often induces oxidative stress and an unbalanced redox system. Oxidative damage causes cell damage and apoptosis and ultimately leads to tissue and organ damage and cell death [6].

Peroxiredoxins (Prxs), such as hydrogen peroxide and alkyl peroxides, are a ubiquitous family of antioxidant enzymes that protect cells from the erosion of reactive oxygen species and participate in a series of cell signaling pathways. Peroxiredoxin is a highly conserved thiol-dependent protein that detoxifies various reactive oxygen species (ROS) and is essential for the maintenance of cellular redox homeostasis [7]. Among the antioxidant proteins, Prx systems were the most recently discovered, involving nonheme peroxidases that catalyze the reduction of H_2_O_2_, peroxynitrite, and alkyl hydroperoxides via Trx and other thiol-containing reducing agents as electron donors. Unlike other peroxidases, the redox process of Prxs did not require the participation of cofactors, and it relied mainly on cysteine with redox activity to exert its effect [8]. At present, Prxs have been identified in almost all organisms, and their expression levels and catalytic rates are relatively high.

On the basis of structural similarity and sequence homology, Prxs have been divided into six subfamilies: AhpC/Prx1, Prx6, Prx5, Tpx, BCP/PrxQ, and AhpE [9]. Based on the catalytic process, Prxs have also been classified into three types: typical 2-Cys, atypical 2-Cys, and 1-Cys Prxs [10]. All types of Prxs relied on cysteine to exert their catalytic effect and referred to as cysteine peroxides (CP), which are usually located in the N-terminal region of Prxs. The catalytic process can be divided into three steps. In the first step, CP is directly responsible for attacking the residues of the oxide, releasing alcohol or water molecules; the second step involves a second cysteine with redox activity, called dissociated cysteine (CR), which is usually located in the C-terminal region, and CR is responsible for attacking the oxidized CP to form a CP-CR disulfide bond. Of course, 1-Cys Prxs lack this step due to the deletion of CR residues. The third step is the reduction and regeneration of oxidized Prxs by thiol-containing electron donors, such as thioredoxins (Trxs), glutathione, or glutathione transferase [11].

In this study, the enzyme active site of the TgAHP1 protein was identified, leading to the determination of alkyl hydroperoxide reductase (Ahp1), which belongs to the Prx5 subfamily and is a cysteine-dependent peroxidase. Our data indicated that TgAHP1 was more sensitive to the tBOOH, and the presence of AHP1 reduced the oxidative stimulation of tBOOH in parasites.

## 2. Materials and Methods

### 2.1. Ethical Statement

The experiments using mice were approved by the Ethics Committee for Animal Experiments of the Laboratory Animal Center of Shandong Agricultural University, China.

### 2.2. Strain Types and Culture Conditions

RH∆Ku80 tachyzoites were cultured in DF-1 cells (a spontaneously immortalized chicken embryo fibroblast cell line) and Vero cells (African green monkey kidney cells) at 37°C and 5% CO_2_. Both DF-1 cells and Vero cells were stored in our laboratory.

### 2.3. Cloning and Expression of Recombinant AHP1 Protein

The gene-specific primers were designed to obtain the TgAhp1 coding sequence (ToxoDB ID: TGGT1_286630) from the tachyzoites of the RH∆Ku80 strain, and pET-Ahp1 was constructed for the prokaryotic protein. Recombinant plasmids pET-Ahp1 (Gly^191^-Arg), pET-Ahp1 (Gly^166^-Arg), and pET-Ahp1 (Gly^166^-Arg, Gly^191^-Arg) were constructed by point mutation method and expressed in *E. coli* BL21 (DE3).

### 2.4. Protein Peroxidase Activity Determination

AHP1, AHP1 (Gly^166^-Arg), AHP1 (Gly^191^-Arg), and AHP1 (Gly^166^-Arg, Gly^191^-Arg) proteins were expressed and diluted to 1 mg/mL, 3 mg/mL, 5 mg/mL, and 7 mg/mL, respectively, and were quickly mixed with the matrix solution (Nanjing Jiancheng). The absorbances were measured at a wavelength of 240 nm at time points of 10 s and 190 s, respectively, in a UV-spectrophotometer (PharmaSpec UV-1700 Shimadzu). CAT activity was calculated by the following formula: [CAT activity (U/gprot) = (ΔA/min × assay volume)/(0.872 × volume of homogenate × Protein concentration)].

### 2.5. Parasite Survival Assays

For all assays, tachyzoites were pretreated with tBOOH (OUHE Technology) diluted to 10^−4^ M with DMEM for 2 h. Parasite plaque, synchronous invasion, and replication assays were performed as described in previous report [12]. The tachyzoites were stained and observed by the immunofluorescence assay (IFA). The number of parasitophorous vacuoles (PV) and the number of host cells were counted in triplicates from three independent biological replicates, and the results were statistically analyzed to assess the invasion efficiency of different strains stimulated by different oxidants. The invasion efficiency was calculated by the number of infected cells/total host cells. One hundred vacuoles were selected, and the number of tachyzoites in each vacuole was counted to evaluate the proliferation efficiency. Real-time images were obtained through a microscope in three independent experiments, and two observers were responsible for statistics and calculation.

### 2.6. qPCR

The tachyzoites of the RH∆Ku80 strain were stimulated with 10^−4^ M tBOOH and 10^−4^ M H_2_O_2_ for 24 h and collected at 2, 8, 14, 20, and 26 h. The RNA extraction kit FastPure® Cell/Tissue Total RNA Isolation Kit (Vazyme Biotech) was used for RNA extraction. cDNA was obtained by reverse transcription using the HiScript® II Q Select RT SuperMix for qPCR (Vazyme Biotech). qPCR detection was performed according to the instructions of ChamQTM Universal SYBR® qPCR Master Mix (Vazyme Biotech), and TgGAPDH was used as a reference gene.

### 2.7. Mice Pathogenicity Test

Seven-week-old female BALB/c mice were purchased from Jinan Pengyue Experimental Animal Breeding Co., Ltd. and bred under the conditions stipulated by the Shandong Provincial Laboratory Animal Affairs Administration. Freshly purified tachyzoites were injected intraperitoneally, and the survival and symptoms of mice were monitored daily.

### 2.8. Intracellular Oxidative Activity Measurement

Intracellular reactive oxygen species (ROS) levels in *T. gondii* were determined using the probe 2′,7′-dichlorofluorescein diacetate (DCF-DA, Solarbio). RH∆Ku80 and ∆Ahp1 tachyzoites were collected, pretreated with tBOOH for different times, and then incubated with 10 *μ*M DCF-DA at 37°C for 20 min. Tachyzoites were washed 3 times with PBS and detected with a fluorescence microplate reader. The excitation and emission wavelengths were 488 and 525 nm, respectively.

### 2.9. Determination of Apoptosis Level

The annexin V/propidium iodide (PI) method was used to detect the apoptosis rate of the parasites, and PI is responsible for staining the nucleus of apoptotic parasites. RH∆Ku80 and ∆Ahp1 tachyzoites were pretreated with tBOOH for different times and washed twice with PBS, and PI was added and then incubated for 10 min. Tachyzoites were observed using a laser scanning confocal microscope, with apoptosis rates calculated for different treatment groups.

### 2.10. Statistical Analysis

Prism 5 (GraphPad Software, Inc., CA, USA) was used for statistical comparison, while Student's *t*-test was used to carry out a two-way analysis of variance. Statistical data are expressed as the mean value of the standard error of the mean (SEM). *P* < 0.05 and *P* < 0.01 were considered statistically significant and extremely significant, respectively.

## 3. Results

### 3.1. Bioinformatic Analysis of *T. gondii* Ahp1

**Figure 1:**
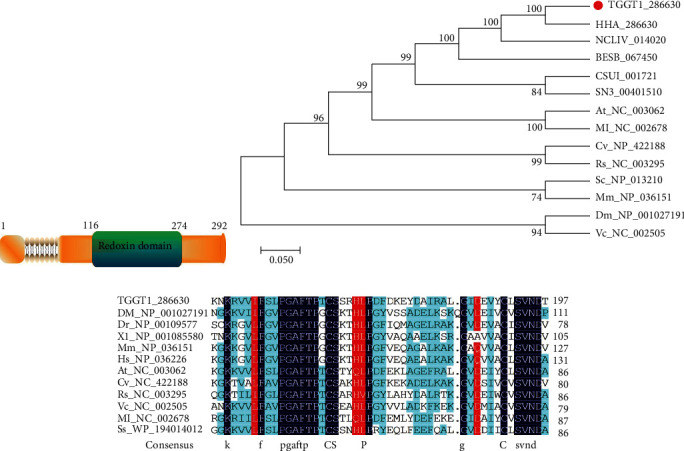
Phylogenetic tree and alignment analysis of AHP1 proteins. (a) According to the Smart Mode online prediction website, the Ahp1 gene of *T. gondii* contains a redoxin domain. (b) A phylogenetic tree of AHP1 proteins in several species. Numbers on nodes indicate bootstrap values, and the scale bar indicates the number of genetic differences at each locus. (c) Alignment of amino acid sequences of AHP1 from *T. gondii* and other model organisms. Amino acid sequences are aligned with Clustal W, identical motifs are colored in black areas, and similar motifs are colored with red characters.

BLAST was performed in the *T. gondii* database based on the yeast AHP1 amino acid sequence to obtain an amino acid sequence with a lower *E* value, and the gene with the accession number TGGT1_286630 (ToxoDB database) was found. The Ahp1 gene of *T. gondii* contains a redoxin domain (Figure 1(a)) and is homologous to *Hammondia hammondi* and *Neospora caninum* through the analysis of a phylogenetic tree (Figure 1(b)). The sequence of TgAHP1 was compared with other species, and two conserved amino acid residues were found at the Cys^166^ and Cys^191^ sites (Figure 1(c)). Based on the sequence alignment results, the AHP1 of *T. gondii* is attributed to an alkyl hydroperoxide with peroxidase activity.

### 3.2. Ahp1 Localized in the Cytoplasm of *T. gondii* Tachyzoites

**Figure 2:**
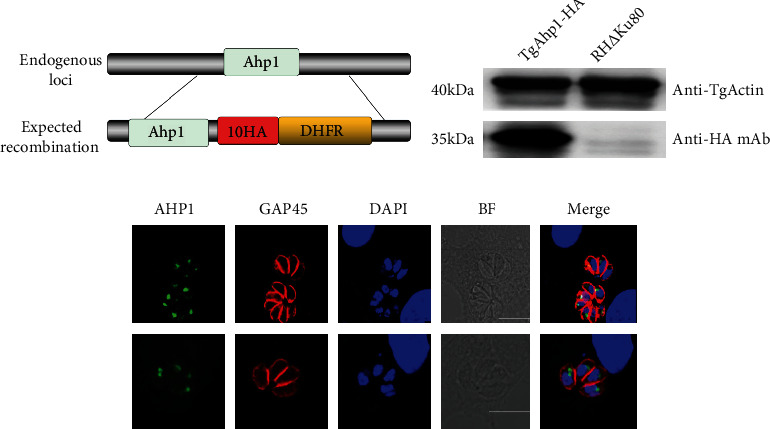
Localization of AHP1 in *T. gondii*. (a) Strategy for constructing HA-tagged fusion-expressing strain. (b) Western blot confirming successful integration of the HA tag. (c) IFA used for TgAHP1 localization observation. TgAHP1 (green) and TgGAP45 (red) in RH∆Ku80 determined the localization. Scale bar, 5 *μ*m. DAPI: 4′,6-diamidino-2-phenylindole, nuclear dye; TgGAP45: gliding associated protein 45; BF: bright field.

To observe the expression of Ahp1 in *T. gondii,* 10 HA tags were fusion expressed in the C-terminal of TgAhp1 (Figures 2(a) and 2(b)). The localization of TgAHP1 in *T. gondii* was determined using an immunofluorescence assay, and the AHP1 protein was found to be presented primarily in a punctate staining pattern anterior to the parasite nucleus (Figure 2(c)).

### 3.3. Enzyme Activity Site Determination of Ahp1 Protein

**Figure 3:**
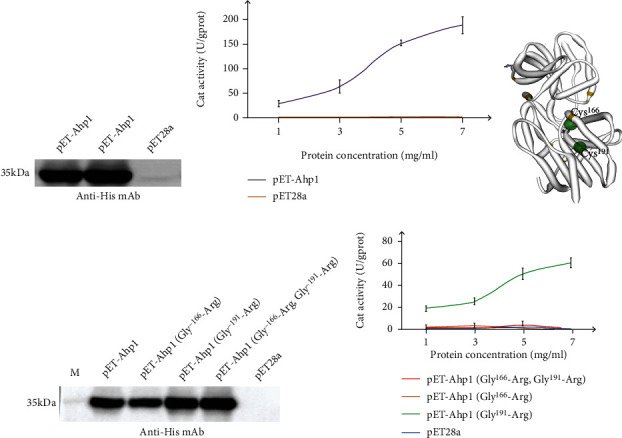
Catalase activity of AHP1 protein in *T. gondii*. (a) Western blot identification of the protein expressed by pET-Ahp1 recombinant plasmid using His monoclonal antibody. (b) CAT activity of TgAHP1 recombinant protein measured at concentrations of 1 mg/ml, 3 mg/ml, 5 mg/ml, and 7 mg/ml. (c) Predicted three-dimensional structure of the TgAHP1 protein. The yellow dots represent cysteines. (d) Western blot identification of the protein expressed by the pET-Ahp1 mutant recombinant plasmids using His monoclonal antibody. (e) CAT activity of TgAHP1 mutant recombinant proteins. Values shown are means ± SEM from three independent experiments (*n* = 3).

The absorbance of the recombinant protein pET-Ahp1 was measured using a peroxidase assay kit under UV spectrophotometer at 10 s and 3 min and 10 s, respectively. The CAT peroxidase activity calculation formula was used to calculate the CAT peroxidase activity under different protein concentrations. The CAT activity increased with increasing protein concentration. This was achieved until the protein concentration was 7 mg/ml, and the CAT activity peaked at 200 U/gprot (Figures 3(a) and 3(b)), indicating the presence of peroxidase activity in TgAHP1. Bioinformatic analysis showed that TgAHP1 belongs to Prx5 with 2-Cys sites. A three-dimensional structure pattern of the TgAHP1 protein (Figure 3(c)) was established to evaluate the disulfide bond between the two cysteines of Cys^166^ and Cys^191^ for redox reactions. Protein mutations of Gly^166^-Arg, Gly^191^-Arg, and (Gly^166^-Arg, Gly^191^-Arg) double mutations were expressed and identified by Western blot (Figure 3(d)). The CAT enzyme activities of these proteins were calculated (Figure 3(e)). The AHP1-Gly^191^-Arg protein still retained peroxidase activity, while the AHP1-Gly^166^-Arg and double mutation Gly^166^-Arg and Gly^191^-Arg proteins lost their enzymatic activities, indicating that Cys^166^ is the key enzyme active site of the AHP1 protein in *T. gondii*.

### 3.4. An TgAHP1 Protein-Sensitive Peroxide of tBOOH

**Figure 4:**
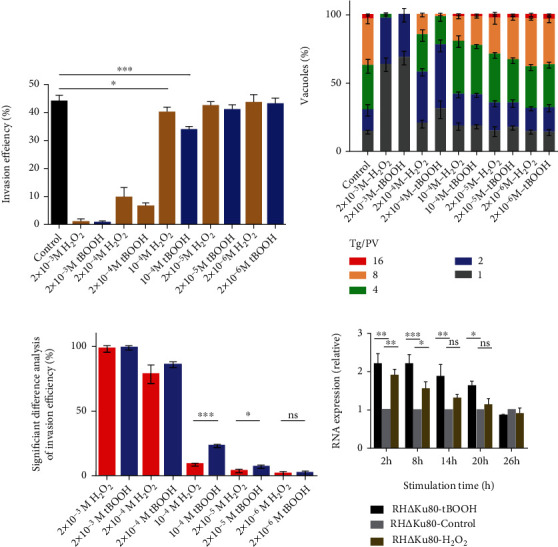
Higher sensitivity of AHP1 to tBOOH than to H_2_O_2_. (a) In vitro detection of parasite invasion. The invading parasites were pretreated with different concentrations of H_2_O_2_ and tBOOH for 2 h. (b) Difference analysis of invasion efficiency treated with different concentrations of H_2_O_2_ and tBOOH. (c) Intracellular replication assay to examine parasite proliferation in vitro. The invading parasites were pretreated with H_2_O_2_ and tBOOH for 2 h, and the experiment was then repeated three times independently (*n* = 3). (d) The RH∆Ku80 strain was stimulated with tBOOH and H_2_O_2_, and the TgAhp1 gene expression was detected at 2, 8, 14, 20, and 26 h. Values shown are means ± SEM from three independent experiments (*n* = 3), each with three replicates. ns, *P* > 0.05 indicates nonsignificant; ^∗^*P* < 0.05; ^∗∗^*P* < 0.01; ^∗∗∗^*P* < 0.001; all by Student's *t*-test.

For screen-sensitive peroxides of *T. gondii*, the RH∆Ku80 strain was treated with different concentrations of H_2_O_2_ and tBOOH, respectively, and the invasion and proliferation assays were performed for RH∆Ku80 strain. The statistical results showed that there was a significant difference in the invasion and proliferation of the strain after treatment with H_2_O_2_ or tBOOH (Figures 4(a)–4(c)), indicating that the RH∆Ku80 strain is more sensitive to peroxides. RH∆Ku80 tachyzoites were treated with tert-butyl hydrogen peroxide (tBOOH) and hydrogen peroxide (H_2_O_2_), and the expressions of the TgAhp1 gene were detected by RT-qPCR at different time points. The transcription levels of TgAhp1 increased when treated with tBOOH and H_2_O_2_, but were higher than that of H_2_O_2_ within 20 h of tBOOH stimulation, indicating that TgAHP1 is more sensitive to tBOOH. After 20 h, the gene expression trend to converge (Figure 4(d)), which may be due to the fact that the host cells and tachyzoites had degraded the peroxides.

### 3.5. Survival Efficiency of Ahp1 Knockout Strain

**Figure 5:**
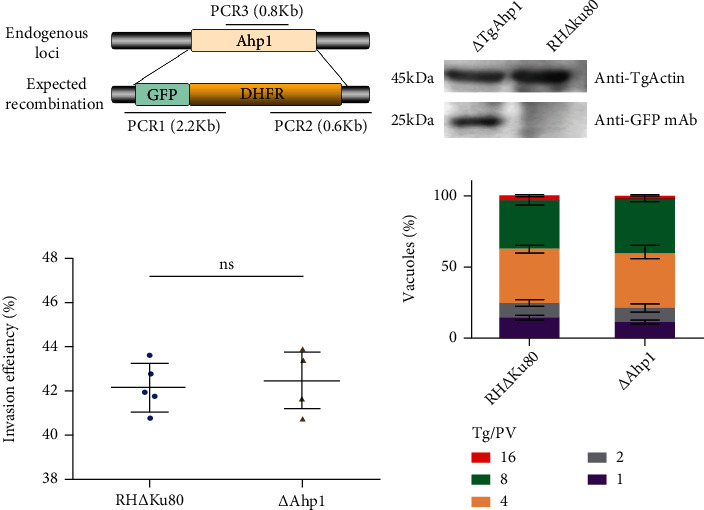
Characterization of an Ahp1 deletion strain. (a) Schematic diagram of Ahp1 knockout in RH∆Ku80 by CRISPR/Cas9-mediated homologous gene replacement. (b) Western blot confirming successful integration of the GFP tag using mouse anti-GFP antibody. (c) Difference invasion efficiency of RH∆Ku80 and ∆Ahp1 strains. (d) Intracellular replication assay for examining RH∆Ku80 and ∆Ahp1 strains proliferation in vitro.

The Ahp1 gene of *T. gondii* was deleted using the CRISPR/Cas9 system (Figure 5(a)), and ∆Ahp1 was verified by Western blot (Figure 5(b)). The knockout of Ahp1 not obviously affected the invasion efficiency of *T. gondii* (Figure 5(c)). Both the ∆Ahp1 strain and the RH∆Ku80 strain contained 4 or 8 parasites at 24 h for each parasitophorous vacuole, and there was no significant difference in statistical analysis (Figure 5(d)), indicating that TgAhp1 is not essential for *Toxoplasma*'s survival efficiency.

### 3.6. Survival Efficiency of Ahp1 Knockout Strain Treated with tBOOH

**Figure 6:**
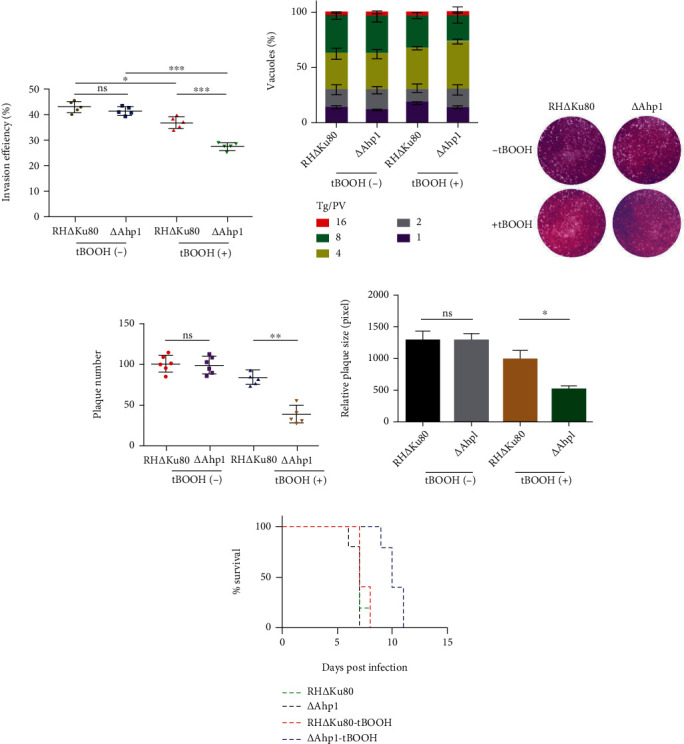
Attenuating the oxidative stimulation effect of tBOOH on *T. gondii* tachyzoites relies on AHP1: (a) Difference invasion efficiency of RH∆Ku80 and ∆Ahp1 strains. The invading parasites were pretreated with 10^−4^ M tBOOH for 2 h. (b) Intracellular replication assay to examine parasite proliferation in vitro. The invading parasites were pretreated with tBOOH for 2 h, and the experiment was then repeated three times independently (*n* = 3). (c) A 7-day plaque assay was performed on ∆Ahp1 and the RH∆Ku80 strains after stimulation with 10^−4^ M tBOOH. (d, e) Statistics of the number of plaques and plaque area of RH∆Ku80 strain and ∆Ahp1 strain formed using ImageJ software. (f) Survival curve of mice infected with the designated strains. The RH∆Ku80 and ∆Ahp1 strains stimulated with 10^−4^ M tBOOH were used to infect Balb/C mice by intraperitoneal injection (500 tachyzoites per mouse, *n* = 10 mice per strain), and the survival conditions of mice were recorded daily.

The RH∆Ku80 and ∆Ahp1 strains were treated with 10^−4^ M tBOOH, respectively, and the invasion and proliferation assays were performed. The statistical results showed that the knocking-out Ahp1 gene weakened the invasion ability of *T. gondii* with 10^−4^ M tBOOH treatment (Figure 6(a)). Under the stimulation of 10^−4^ M tBOOH, the RH∆Ku80 strain had significantly more octoploids than the ∆Ahp1 strain (Figure 6(b)), inferring that the RH∆Ku80 strains has obvious proliferation. Plaque numbers and sizes were determined for 7 days. By statistical analysis, no significant difference could be observed in the size of the plaque in the untreated control group (Figure 6(c)). More plaques and a larger plaque area (Figures 6(d) and 6(e)) were formed by the RH∆Ku80 strain than ∆Ahp1 strain after treatment with 10^−4^ M tBOOH. No significant changes were found in the mice pathogenicity test between the ∆Ahp1 and RH∆Ku80 strains (Figure 6(f)). However, tBOOH reduced the pathogenicity of ∆Ahp1 strains in mice (Figure 6(f)). These indicate that the presence of the Ahp1 gene weakens the oxidative stimulation effect of tBOOH on tachyzoites. Therefore, tBOOH may be related to the redox reaction of TgAHP1.

### 3.7. Induction of Excessive ROS and Apoptosis in Ahp1 Knockout Strains by tBOOH

**Figure 7:**
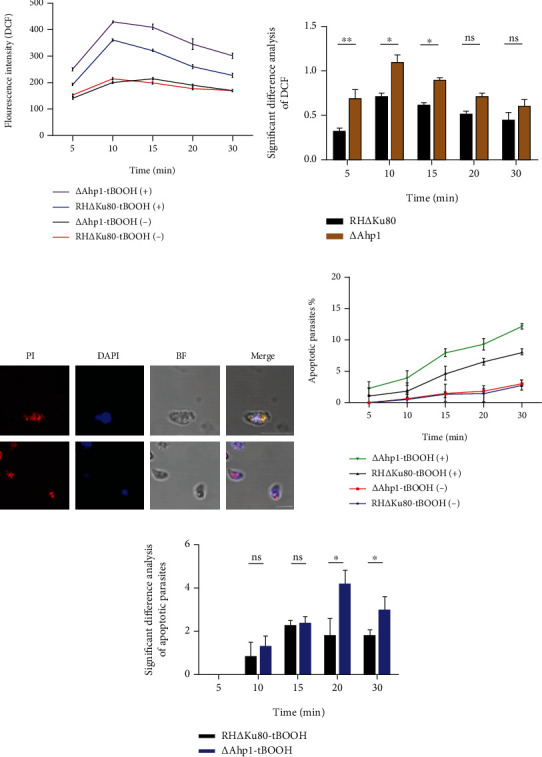
tBOOH induced an increase in ROS levels and apoptosis in Ahp1-deficient parasites. (a) tBOOH induced ROS in the RH∆Ku80 and ∆Ahp1 strains. (b) Statistical analysis showed that the ∆Ahp1 strain produced significantly higher ROS than the RH∆Ku80 strain before 15 min. (c) Two lines real-time images of parasite apoptosis were observed through a fluorescence microscope in different visual field. (d) Statistical calculation of the apoptosis rate of ∆Ahp1 and RH∆Ku80 strains under tBOOH stimulation. The numbers of apoptotic tachyzoites in each visual field were counted to obtain the apoptosis rate of the different treatment groups. (e) tBOOH significantly induced apoptosis in the ∆Ahp1 strain after 15 min (*P* < 0.05). The apoptosis rates were calculated: [∆Ahp1 − tBOOH(+)−∆Ahp1 − tBOOH(−)]/∆Ahp1 − tBOOH(−) and [RH∆Ku80 − tBOOH(+) − RH∆Ku80 − tBOOH(−)]/RH∆Ku80 − tBOOH(−). DCF: dichlorodihydrofluorescein; PI: propidium iodide. Scale bar, 5 *μ*m.

Reactive oxygen levels were detected in parasites stimulated with tBOOH, and a more rapid increase in DCF was observed in the ∆Ahp1 strain compared to the RH∆Ku80 strain. Until 15 min of stimulation, the trend towards equilibrium (Figures 7(a) and 7(b)) revealed that reactive oxygen species accumulated more in the ∆Ahp1 strain. The possibility of knocking-out the Ahp1 gene promotes the production of ROS in the parasites, which are related to the survival of the parasites in the host cells. Excessive ROS often induce oxidative stress in parasites, resulting in oxidative damage and possibly apoptosis. The tBOOH-induced tachyzoites of RH∆Ku80 and ∆Ahp1 strains were observed with a fluorescence microscope, and the apoptotic tachyzoites were stained red by PI (Figure 7(c)). The number of apoptotic tachyzoites was counted, and it was found that the apoptosis rate of ∆Ahp1 tachyzoites stimulated with tBOOH was significantly higher than that of RH∆Ku80 tachyzoites (Figures 7(d) and 7(e)), indicating that tBOOH tends to cause apoptosis in Ahp1 knockout strain.

## 4. Discussion

Several reports have shown that parasites such as *T. gondii* require an effective antioxidant system to maintain the critical balance between antioxidants and prooxidants for their survival in the host cell. The use of dichlorofluorescein diacetate (an endogenous oxidative stress detector) to verify that walnut wood ketone and phenazine methyl sulfate are potentially toxic to *T. gondii* without affecting host cells suggests that *T. gondii* is susceptible to oxidative damage caused by the breakdown of redox balance [13]. Through the phylogenetic analysis of peroxidases from different species, TgAhp1 was similar to HHA_286630 of *Harmon coccidia* and NCLIV_014020 from *Neospora,* indicating that the peroxidase AHP1 not only exists in *T. gondii* but also in other protozoa.

*T. gondii* possesses three types of peroxidases, including one type of 1-Cys peroxidase and two types of 2-Cys peroxidases [14]. Proteins of the Prx family reduced peroxides at the expense of their free cysteine residues, thereby forming disulfide bonds or peroxydisulfuric acid [15]. Studies have shown that disulfide bonds can be formed between the two cysteines of Ahp1, which play a corresponding role in the enzymatic activity. The amino acid sequence of TgAHP1 includes six cysteines, and multiple sequence alignments showed that the Cys^166^ and Cys^191^ sites are conserved with Prx5 subfamily proteins in several species of fungi and bacteria [16]. The results of our enzyme activity assay showed that no peroxidase activity was detected in the TgAHP1 protein when Cys^166^ was mutated. However, the TgAhp1 protein still retained peroxidase activity even when Cys^191^ was mutated, indicating that Cys^166^ is one of the active sites of the enzyme, whereas Cys^191^ is not. Therefore, the oxidation-reduction reaction occurring in TgAHP1 depends on the Cys^166^ site; nevertheless, based on the present results, the redox process of TgAHP1 (relying on 1-Cys or 2-Cys) is still uncertain.

The reactive oxygen decomposition system is a powerful cytoplasmic antioxidant system in *T. gondii*, which helps the parasite to cope with the oxidative stress encountered in the intracellular parasitic life [17]. As a peroxidase, Ahp1 is a receptor for alkyl hydroperoxides (including lipid peroxides) and is responsible for resisting unsaturated lipids [16]. *Saccharomycetes* are sensitive to tert-butyl hydroperoxide with Ahp1 knocking out [18], and yeast Ahp1 is considered an important cytoplasmic thioredoxin-dependent alkyl hydroperoxide reductase, which is involved in oxidative stress [19]. Thess reports are consistent with TgAHP1 for tBOOH sensitivity. The presence of the TgAhp1 gene may be associated with a reduction in hydroperoxides, which in turn reduces the oxidative stimulation of *T. gondii*. The forma tion of disulfide bridges was progressively prevented when the quantity of alkyl hydroperoxides was high and when the enzyme was incubated with all the components required to sustain its catalytic cycle [20]. Knocking out the TgAhp1 gene led to the accumulation of reactive oxygen species in the parasite and induced apoptosis, thus confirming this speculation. TgAhp1 may play a certain protective role against oxidative damage to DNA. Parasites survive in a redox equilibrium state under normal circumstances; however, an imbalance occurs when the concentration of free radicals increases due to internal or external factors [21], which can lead to peroxidation of biological macromolecules, such as oxidative DNA damage, and then affect the growth and survival of cells [22]. The comet assay, also known as single cell gel electrophoresis (SCGE), is a technique for detecting DNA damage [23], which shows DNA damage by calculating the migration rate of parasite DNA in the gel [18]. However, in our results, it was difficult to accurately determine the oxidative protection of the TgAHP1 protein on parasite DNA (date unpublished) by the comet assay. The relevance of TgAHP1 to DNA protective function needs further confirmation.

Some reports have shown that knocking out the Ahp1 gene enhances oxidant damage to somatic cells. In *Saccharomyces cerevisiae* and some bacteria, the Ahp1 protein participated in oxidative stress in the cytoplasm [24], which is same to TgAHP1 and located in the cytoplasm of *T. gondii*. Catalase is the characteristic marker enzyme of peroxisomes and is highly conserved across species. Regardless of the CAT's importance in detoxifying H_2_O_2_, this enzyme is lacking in most pathogenic protozoans [20]. The deduced amino acid sequence of *T. gondii* catalase has typical features of eukaryotic catalases [4, 25]. A cytosolic catalase can act on the detoxification of the majority of host born peroxides due to its high substrate turnover [4, 26].

The Urm1 ubiquitin-like system of *Drosophila* is one of the oldest protein functional regulatory systems in eukaryotes, which is related to cell growth and development, sulfur transport, nitrogen synthesis, and antioxidative stress [27]. Several reports have shown that this system specifically regulates the activity of Ahp1 (Prx5 family) and affects the antioxidant response in *Drosophila* [28]. Members of the Urm1 family had conserved functions in the oxidative stress response, and Urm1 target proteins had been identified from yeast, fungi, *Drosophila*, and human cells [29]. In addition, Ahp1 forms a dimer with a complete redox center that is closely related to Urm1 receptor activity in the uric acidification of *S. cerevisiae* [30]. When a mutation occurs in Ahp1, two residues near the redox active center will greatly reduce the level of urea ification without compromising the protective effect of tBOOH [31]. There is a possibility that the residue Lys of TgAhp1 can covalently bind to the ubiquitin-related modifier Urm1 to regulate the oxidative stress response. Studies from our laboratory have shown that the Urm1 ubiquitin system exists in *T. gondii* (date unpublished), and the TgUrm1 ubiquitin regulatory system may also regulate TgAhp1, thereby affecting the oxidative stress of *T. gondii*. Currently, TgURM1 and TgAHP1 have been confirmed as a pair of interacting proteins (date unpublished), and other works are in progress.

Substrates of Ahp1 also have a deleterious effect on the enzyme activity. The decrease in enzyme activity is linked to the irreversible modification of one of the two cysteines involved in the disulfide-linked dimerization process. The antioxidative stress response of TgAHP1 depends on the formation and breaking of disulfide bonds. The inactivation of Ahp1 during oxidative stress may have important biological implications.

## Data Availability

The data used to support the findings of this study are included within the article.
